# *Pseudomonas aeruginosa* Transmigrates at Epithelial Cell-Cell Junctions, Exploiting Sites of Cell Division and Senescent Cell Extrusion

**DOI:** 10.1371/journal.ppat.1005377

**Published:** 2016-01-04

**Authors:** Guillaume Golovkine, Eric Faudry, Stéphanie Bouillot, Sylvie Elsen, Ina Attrée, Philippe Huber

**Affiliations:** 1 University Grenoble Alpes, Grenoble, France; 2 CNRS, ERL5261, Grenoble, France; 3 CEA, iRTSV-BCI, Grenoble, France; 4 INSERM, U1036, Grenoble, France; University of Washington, UNITED STATES

## Abstract

To achieve systemic infection, bacterial pathogens must overcome the critical and challenging step of transmigration across epithelial barriers. This is particularly true for opportunistic pathogens such as *Pseudomonas aeruginosa*, an agent which causes nosocomial infections. Despite extensive study, details on the mechanisms used by this bacterium to transmigrate across epithelial tissues, as well as the entry sites it uses, remain speculative. Here, using real-time microscopy and a model epithelial barrier, we show that *P*. *aeruginosa* employs a paracellular transmigration route, taking advantage of altered cell-cell junctions at sites of cell division or when senescent cells are expelled from the cell layer. Once a bacterium transmigrates, it is followed by a cohort of bacteria using the same entry point. The basal compartment is then invaded radially from the initial penetration site. Effective transmigration and propagation require type 4 pili, the type 3 secretion system (T3SS) and a flagellum, although flagellum-deficient bacteria can occasionally invade the basal compartment from wounded areas. In the basal compartment, the bacteria inject the T3SS toxins into host cells, disrupting the cytoskeleton and focal contacts to allow their progression under the cells. Thus, *P*. *aeruginosa* exploits intrinsic host cell processes to breach the epithelium and invade the subcellular compartment.

## Author Summary

In normal situations, the mucosae constitute efficient barriers against the invasion of opportunistic pathogens. The bacteria inducing nosocomial infections take advantage of pre-existing pathological situations to cross the epithelium and spread in deeper tissues. The conditions on the host side permitting transmigration and the combination of virulence factors used by the bacteria to transmigrate are mostly speculative. Here, we studied the transmigration process of *Pseudomonas aeruginosa*, a bacterium causing hospital-acquired acute and chronic infections. We found that bacteria exploits weakened cell-cell junctions of the epithelium, such as those generated at sites of cell division or when dying cells are extruded from the cell layer, to breach the cell layer, using specific virulence factors and motility appendages.

## Introduction

*Pseudomonas aeruginosa* is a major opportunistic bacterial pathogen associated with nosocomial infections. It is the principal agent responsible for mortality in cystic fibrosis patients and one of the main bacteria linked to hospital-acquired infections, particularly infections sustained after the placement of therapeutic devices such as ventilators, blood or urinary catheters. With acute infections, *P*. *aeruginosa* can disseminate from the initial site of infection across tissue barriers to induce bacteremia and systemic infection [1].

*P*. *aeruginosa* is present in the environment and in the human digestive and respiratory tracts, but healthy people are resistant to infection despite its arsenal of virulence factors. This resistance suggests that the epithelial barriers together with the action of immune cells constitute efficient protection mechanisms. Indeed, several groups have shown that the apical domain of epithelial cells, when assembled in monolayers, is refractory to intoxication by *P*. *aeruginosa*, which could explain why people with uninjured tissues are resistant to infection [2–6]. Consistent with these findings, epithelial tight junctions have been reported to play a role in the protection against *P*. *aeruginosa* invasion [5, 7].

*P*. *aeruginosa*’s virulence factors include exotoxins which most strains produce and inject directly into the cytoplasm of target cells through their type 3 secretion system (T3SS), a syringe-like apparatus common to many Gram-negative pathogenic bacteria [8, 9]. The T3SS effectors (ExoS, T, Y and U) are considered to be *P*. *aeruginosa'*s most potent toxins, because strains not expressing T3SS are much less virulent both in the clinic and in animal models of infection [10–14]. The majority of clinical strains secrete Exo S, T and Y, all of which disrupt the cytoskeleton to cause cell retraction, or cell rounding, eventually leading to cell detachment; ExoU induces rapid necrotic cell death [9].

*P*. *aeruginosa* also has type 4 pili (hereafter called pili) and a flagellum at its surface. These structures are used for movement due to twitching and swimming motions, respectively. A third motion type, called swarming, allows motility across semi-solid surfaces, and is promoted by the flagellum, the pili and bacterial surfactants [15]. In animal models of infection, pili and the flagellum are both required for full virulence [16, 17]. Pili are composed of retractile fibers, and—in addition to their role in motility—serve to attach the bacterium to host cells in preparation for toxin injection through the T3SS [6, 18]. Pili have also been shown to preferentially interact with the cells’ basolateral domain [6, 18, 19].

Recently, Heiniger *et al*. [6] demonstrated that pili and T3SS are required for *P*. *aeruginosa* to cross an epithelial barrier, potentially by interacting with exposed basolateral surfaces. These results suggest a paracellular route of transmigration. However, these authors analyzed the infection process using fixed samples, and provided no information on the sequence of events leading to initial barrier disruption and bacterial transmigration.

Internalization of *P*. *aeruginosa* in non-phagocytic cells is known to be facultative. In epithelial monolayers, it has been proposed that bacterial uptake involves the transformation of a sub-domain of the apical cell surface into a basolateral-like domain [20, 21], which would indicate a transcellular route of transmigration. However, the whole transmigration process and the conditions required for its achievement have never been completely characterized.

In this paper, we investigated *P*. *aeruginosa* transmigration across polarized epithelial monolayers using real-time 3-dimensional microscopy. Our results indicate that bacteria transmigrated using paracellular routes at very specific points: sites of cell division or cell death, where intercellular junctions are temporarily discontinuous. No internalization was observed, thus precluding a transcellular transmigration route. Time-course experiments showed that bacteria induced cell rounding only once they had gained access to the cells’ basolateral domain. Bacterial progression below the cells was possible after disruption of the cytoskeleton and focal contacts upon toxin injection, and the entire process was shown to require functional pili, the T3SS and the flagellum.

## Results

### Spatio-temporal visualization of *P*. *aeruginosa* transmigration across epithelial monolayers

**Fig 1:**
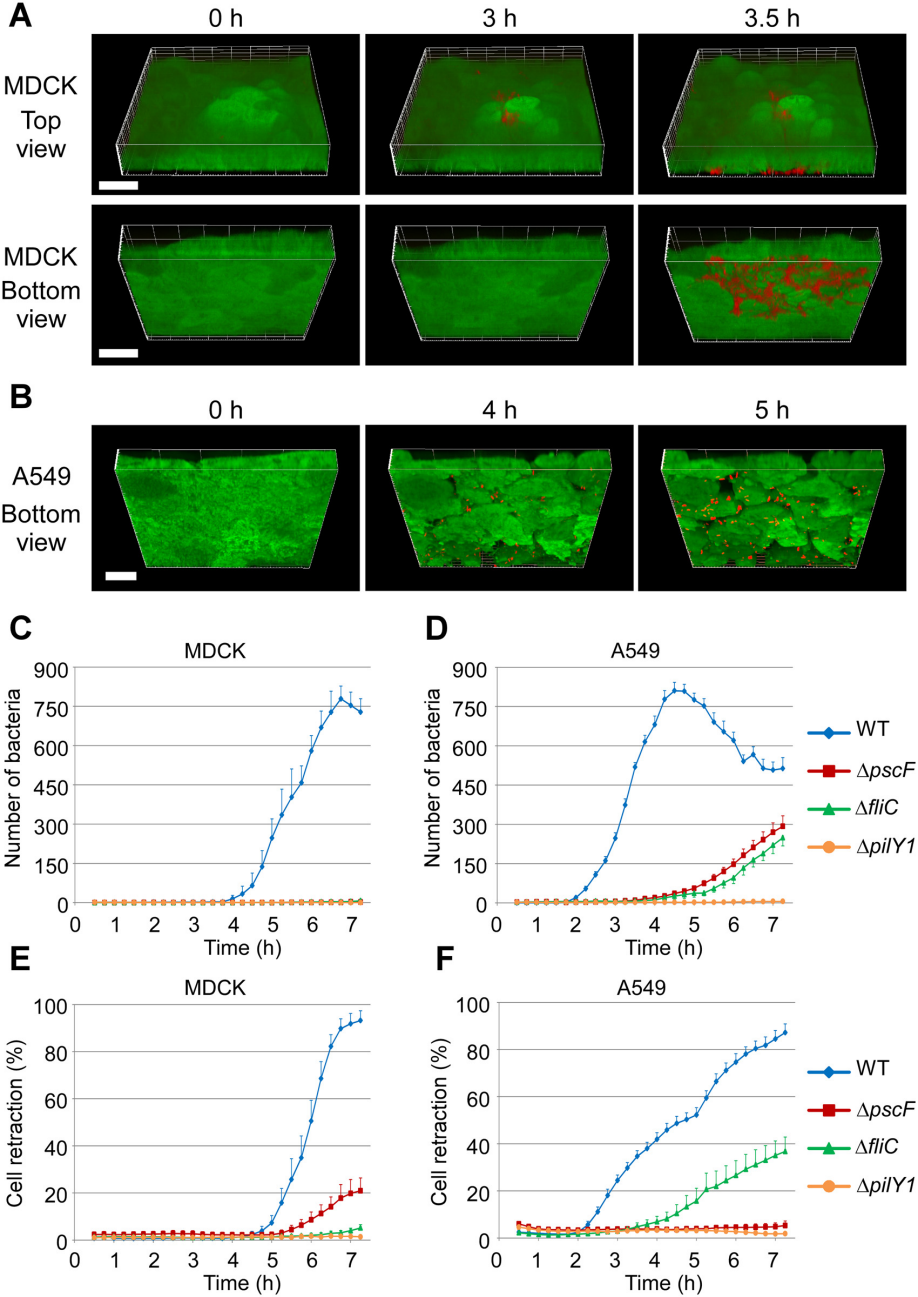
*P*. *aeruginosa* crossing through epithelial barriers. (**A**) Reconstituted 3D images of WT bacteria (red) crossing through MDCK monolayers (green). Selected frames from S1 Movie. Transmigration was observed simultaneously from the apical surface (Top view) and from the basal compartment (Bottom view) at indicated times post infection. (**B**) Similar experiment with A549 monolayer. Frames were selected from S2 Movie. 3D-reconstitution was performed from 30 and 40 confocal z-planes for A549 and MDCK, respectively. Scale bars: 15 μm. (**C,D**) The averaged number (+ SEM) of bacteria located in the basal compartment are plotted against time post infection of MDCK (**C**) and A549 monolayers (**D**) (n = 10 fields per condition). Cells were infected by either the wild type (WT), or mutants lacking T3SS (Δ*pscF*), flagellum (Δ*fliC*) or pili (Δ*pilY1*). Images showing the transmigrated bacteria for each strain at different time points are presented for MDCK in S2 Fig (**E,F**) Cell retraction was measured on the same datasets as in (**C,D**) for MDCK (**E**) and A549 (**F**), using z-projections of the 5 lowest confocal images.

In this paper, we investigated *P*. *aeruginosa* (strain CHA labeled with mCherry) transmigration across epithelial monolayers (EGFP-labeled confluent MDCK cells). MDCK monolayers are formed by polarized cells with continuous tight junctions (S1A Fig) and constitute an established cellular model for the study of *P*. *aeruginosa* virulence [4, 22, 23]. After exposure to *P*. *aeruginosa*, 3D confocal microscopy images of bacteria and MDCK cells were acquired for 7 hours at different locations on the cell monolayer. Bacteria were seen to transmigrate at specific sites and to propagate radially below the cell layer (Fig 1A and S1 Movie). Transmigration was consistently seen to occur via a paracellular route (i.e., through cell-cell junctions), first by a single bacterium, rapidly followed by a cohort of bacteria, reminiscent of the "pack swarming" observed when *P*. *aeruginosa* infects macrophages [24]. In contrast, A549 cell layers, formed by non-polarized cells with discontinuous tight junctions (S1B Fig), were penetrated at many different points, also via paracellular routes (Fig 1B and S2 Movie). Thus, intercellular junctions appear to be key to restricting *P*. *aeruginosa* transmigration.

To determine the rate of penetration, the number of bacteria in the basal compartment was quantified at different time points, in 10 microscopy fields per condition, and was expressed as a function of hours-post-infection (hpi) (Fig 1C and 1D). Transmigration across MDCK layers started at approximately 3.5 hpi and reached a peak at 6.5 hpi (Fig 1C; selected images are shown in S2A Fig). In contrast, transmigration across A549 cells started at 2 hpi and reached a peak at 4 hpi (Fig 1D), confirming the permissiveness of A549 cell layers. After bacterial invasion, the cells detached from the coverglass and bacteria were released back into the supernatant, which explains why the number of bacteria decreased in the basal compartment at later time points (Fig 1C and 1D). These results were confirmed with *P*. *aeruginosa* strain PAK applied to MDCK cell layers (S2B Fig).

We next wished to determine which bacterial components were involved in the transmigration process. We therefore used real-time microscopy to examine whether isogenic mutants defective in the T3SS (Δ*pscF*), the flagellum (Δ*fliC*) or pili (Δ*pilY1*) could transmigrate across epithelial layers. None of the mutants transmigrated across MDCK layers (Fig 1C). In contrast, with A549 cell layers transmigration was abolished by pili deficiency, whereas lack of the flagellum or the T3SS was less detrimental (Fig 1D), although it did still greatly reduce the transmigration efficiency. Thus, only pili are absolutely required for transmigration with a permissive epithelium, whereas all three factors are required to cross a polarized epithelium.

The three mutant strains were also used to study the link between bacterial transmigration and cell retraction, a typical effect of ExoS or ExoT injection [25–28]. MDCK cells retracted upon infection with the wild-type strain, while the pili mutant did not induce cell retraction; a slight retraction was observed with the T3SS and flagellum mutants at late time points (Fig 1E and S2A Fig). However, this mild effect was not typical of T3SS-induced cell retraction, rather it was related to intercellular junction remodeling (arrowheads in S2A Fig). A549 cells also retracted when infected with the wild type strain, and to a lesser extent when infected with the flagellum-deficient strain. The T3SS and pili mutants induced no retraction (Fig 1F), indicating that junction remodeling did not occur in this model.

Altogether, these results demonstrate that transmigration, when possible, consistently occurs at cell-cell junctions. In epithelial layers with sealed junctions, mimicking *in vivo* epithelia, pili, the T3SS and the flagellum were all required to initiate transmigration. These results support those of previous studies showing that T3SS effectors and pili are required for efficient penetration of epithelial monolayers [6, 18].

### Bacterial transmigration occurs at sites of host cell division or cell death

**Fig 2:**
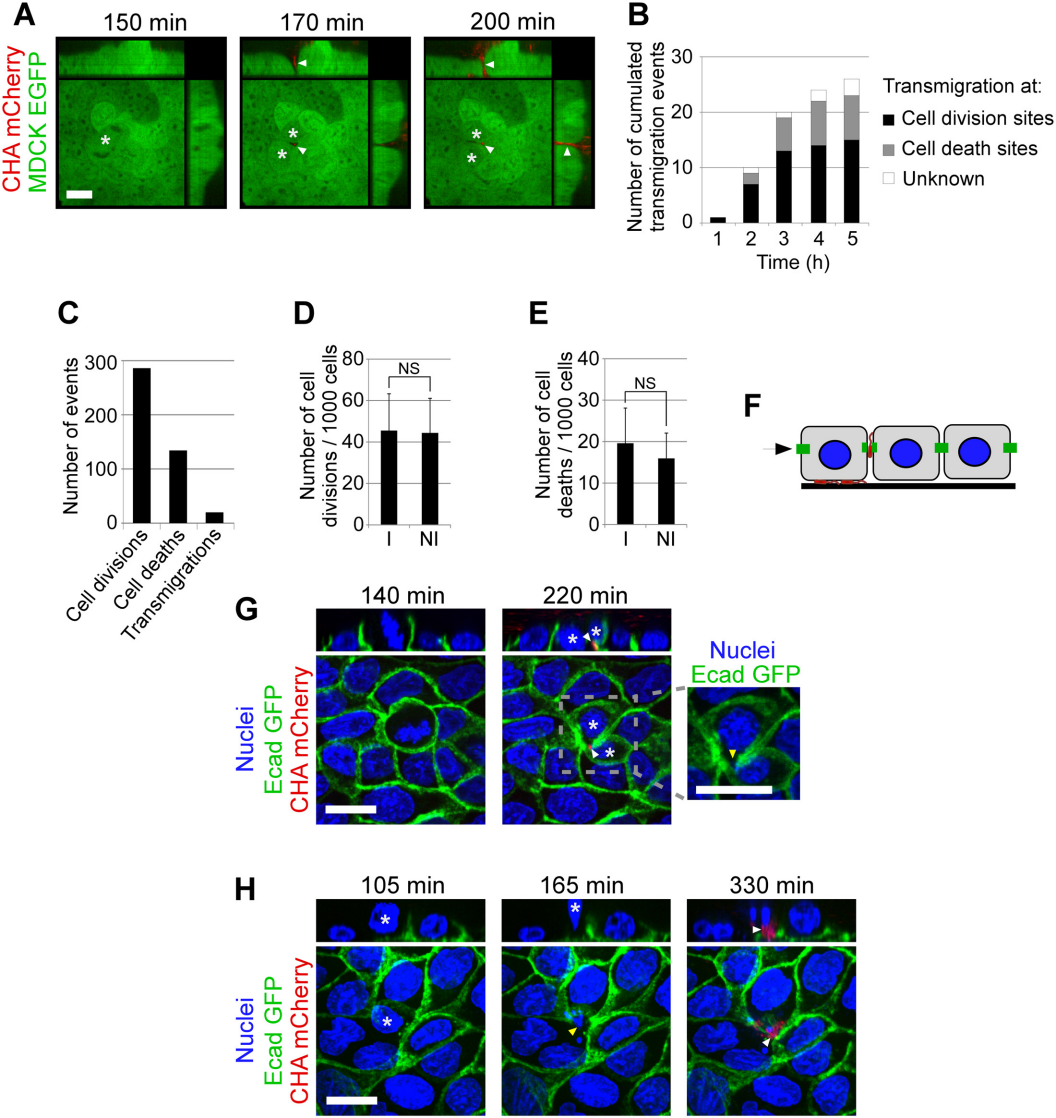
Transmigration sites in epithelial monolayers. (**A**) Confocal images in (x,y), (x,z) and (y,z) showing the transmigration of bacteria (red) in a cleft (arrowhead) formed between two dividing MDCK cells (*). Scale bar: 20 μm. (**B**) The total number of transmigration sites was scored on 48 fields, representing 28,000 cells, and were plotted against time. Transmigration occurred at cell division, cell death or at uncharacterized events (Unknown), as indicated. (**C**) The total numbers of cell division, cell death and the transmigration sites were scored in the first 3 hours of infection. (**D**) The number of dividing MDCK cells was determined in 24 fields for 3 hours in infected (I) and non-infected (NI) conditions. (**E**) The number of dying cells were counted on the same dataset. N.S., not significant according to Student's test. (**F**) Diagram showing the experimental model used in (**G,H**). MDCK monolayers expressing Ecad-EGFP and labeled with Hoechst were infected with CHA-mCherry. (**G**) Confocal images in (x,y) and (x,z) showing a bacterium (white arrowhead) penetrating the intercellular junction at a site of interrupted E-cadherin labeling (yellow arrowhead), between two daughter cells (*), following division. Scale bar: 15 μm. Selected frames from S3 Movie. (**H**) Confocal images in (x,y) and (x,z) showing a bacterial penetration (arrowhead) at intercellular junctions at site of senescent cell extrusion (*). Scale bar: 15 μm. Selected frames from S4 Movie.

We next wished to determine the factors initiating the transmigration process. As MDCK monolayers are a good model of physiological epithelia, we used them for these further studies. Close examination of all the transmigration events occurring with MDCK monolayers revealed that bacteria can use very small gaps ("clefts") formed between daughter cells upon cell division (Fig 2A). Like in Fig 1A, transmigration first involved a single bacterium penetrating the cleft, rapidly followed by a group of bacteria attracted to the transmigration site (Fig 2A). Less frequently, bacteria were also observed to transmigrate at sites where senescent cells had been expelled from the monolayer (see below). Transmigration sites were observed for 5 hours on 48 microscopic fields, representing 28,000 cells, and were classed,using nucleus labeling with vital Hoechst, as sites of cell division, cell death or unknown—when the phenomenon was ambiguous (Fig 2B). The results show that cell division and cell death are the major, probably only, permissive events allowing bacterial transmigration. Transmigration occurred mainly and more rapidly at cell division sites, and was less frequent and occurred later at cell extrusion sites (Fig 2B).

Transmigration remained a rare event, and many sites of cell division or death were not exploited by bacteria for transmigration (Fig 2C), suggesting that other, as yet undiscovered, parameters may contribute to the transmigration process. To determine whether the presence of bacteria alters cell division or cell senescence rates in the MDCK monolayer, we compared the number of dividing and dying cells in uninfected and infected monolayers until 3 hpi, i.e., before massive intoxication with the T3SS toxins (Fig 2D and 2E). In this time frame, the presence of bacteria neither affected cell division nor senescence rates, indicating that bacterial transmigration at specific sites is an opportunistic rather than an instrumental event.

The fact that specific cellular processes are linked to transmigration events suggests particularities of the intercellular junctions at these positions. We therefore used MDCK cells expressing E-cadherin-EGFP to more precisely analyze the cell-cell junctions during cell division and cell death. During cell division, these cells revealed minute interruptions in E-cadherin staining between dividing cells, where bacteria could penetrate the epithelial layer (Fig 2F and 2G and S3 Movie). In the case of cell death, senescent cells were extruded from the monolayer while adjacent cells formed new junctions below the cell being expelled, as previously reported [29]. Like with cell division, gaps in E-cadherin-EGFP labeling were observed in the newly-formed junctions (Fig 2H and S4 Movie) and bacteria availed of these to gain access to the sub-epithelium. Thus, *P*. *aeruginosa* exploits natural weaknesses in the epithelial monolayer to gain access to the basal domain.

### T3SS-dependent intoxication allows bacterial progression under the cells

**Fig 3:**
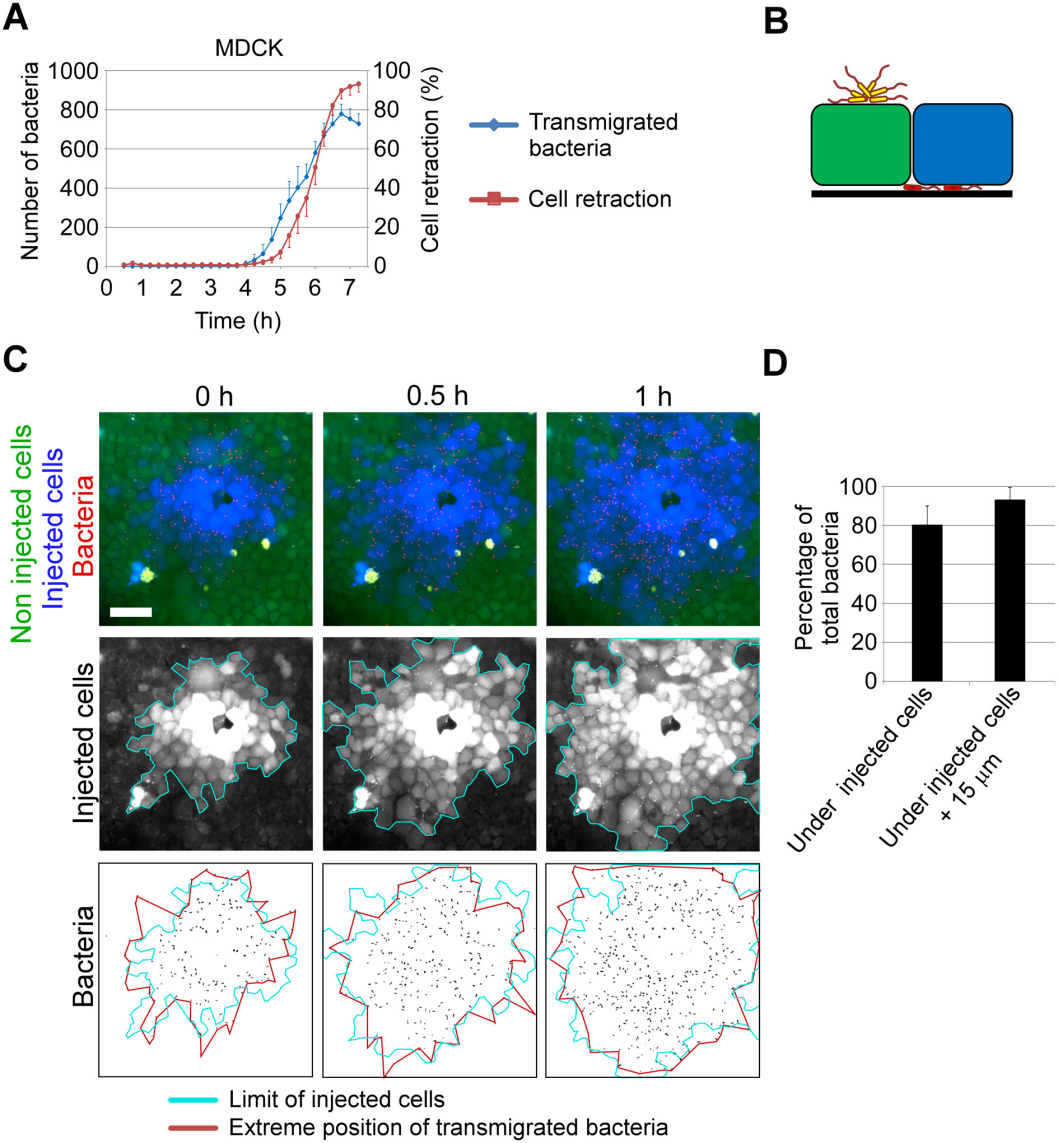
Visualization of T3SS toxin injection in epithelial monolayers. (**A**) The bacterial transmigration kinetics is shown as in Fig 1C and compared to cell retraction (Fig 1D), owing to toxin injection, measured on the same dataset (means + SEM, n = 10 fields). Cell retraction was consistently delayed compared to bacterial transmigration in each field. (**B**) Diagram showing the experimental system used in (**C**) to monitor T3SS toxin injection: bacteria injecting an ExoS-Bla chimeric toxin were used to infect MDCK monolayers loaded with CCF2, a bifluorescent Bla substrate (see SI Materials and Methods). Once CCF2 is cleaved by ExoS-Bla, fluorescence is shifted from green to blue. Bacterial aggregates at apical surface are represented in yellow, and bacteria in the basal compartment are in red. (**C**) Top: merged wide-field images at different time points post-CCF2 loading, centered at a transmigration site. Scale bar: 50 μm. Images are selected frames from S5 Movie. Middle: images showing only the blue channel (injected cells). A blue line indicates the external limit of injected cells. Bottom: images showing the bacteria in the basal compartment. The red line represents the outermost bacteria positions and the blue line is as above. (**D**) Histogram showing the percentage of bacteria within the injected cell surface in 12 fields at 4 h (MOI 30), and within the injected cell surface dilated of 15 μm on each side.

To establish a sequence of events in the transmigration process, we observed T3SS-induced cell retraction together with bacterial accumulation in the basal compartment using time-lapse microscopy. The curve representing average cell retraction (Fig 3A) is shifted to the right, or later time points, compared to the average transmigration curve. This shift was consistently observed for each location analyzed. Thus, cell retraction appears to occur when bacteria have already gained access to the basal compartment.

To visualize toxin injection, we used a previously reported system [14, 30], in which bacteria secrete an ExoS toxin-ß-lactamase (Bla) fusion protein (ExoS-Bla), and MDCK cells are loaded with CCF2, a bifluorescent ß-lactamase substrate. With this system, toxin injection is revealed by cleavage of CCF2 by ExoS-Bla, shifting cellular fluorescence from green to blue (Fig 3B). Blue fluorescence was only detected at and around transmigration sites, and was particularly intense in cells close to the entry site (Fig 3C and S5 Movie). The fluorescence shift expanded radially, mirroring bacterial propagation under the monolayer. The leading edges of bacterial migration consistently correlated with the positions of the outermost injected cells (Fig 3C). The percentage of bacteria in the basal compartment within areas containing infected cells (i.e., blue cells) was 80%; this percentage increased to 93% when the areas were increased by 15 μm (Fig 3D), confirming the correlation between bacterial progression under cells and toxin injection. These observations strongly suggest that bacteria located in the basal compartment inject toxins into cells to allow their progression. Bacterial aggregates were also noted close to the cell surface, but their positions did not correspond to transmigration sites (Fig 3C).

**Fig 4:**
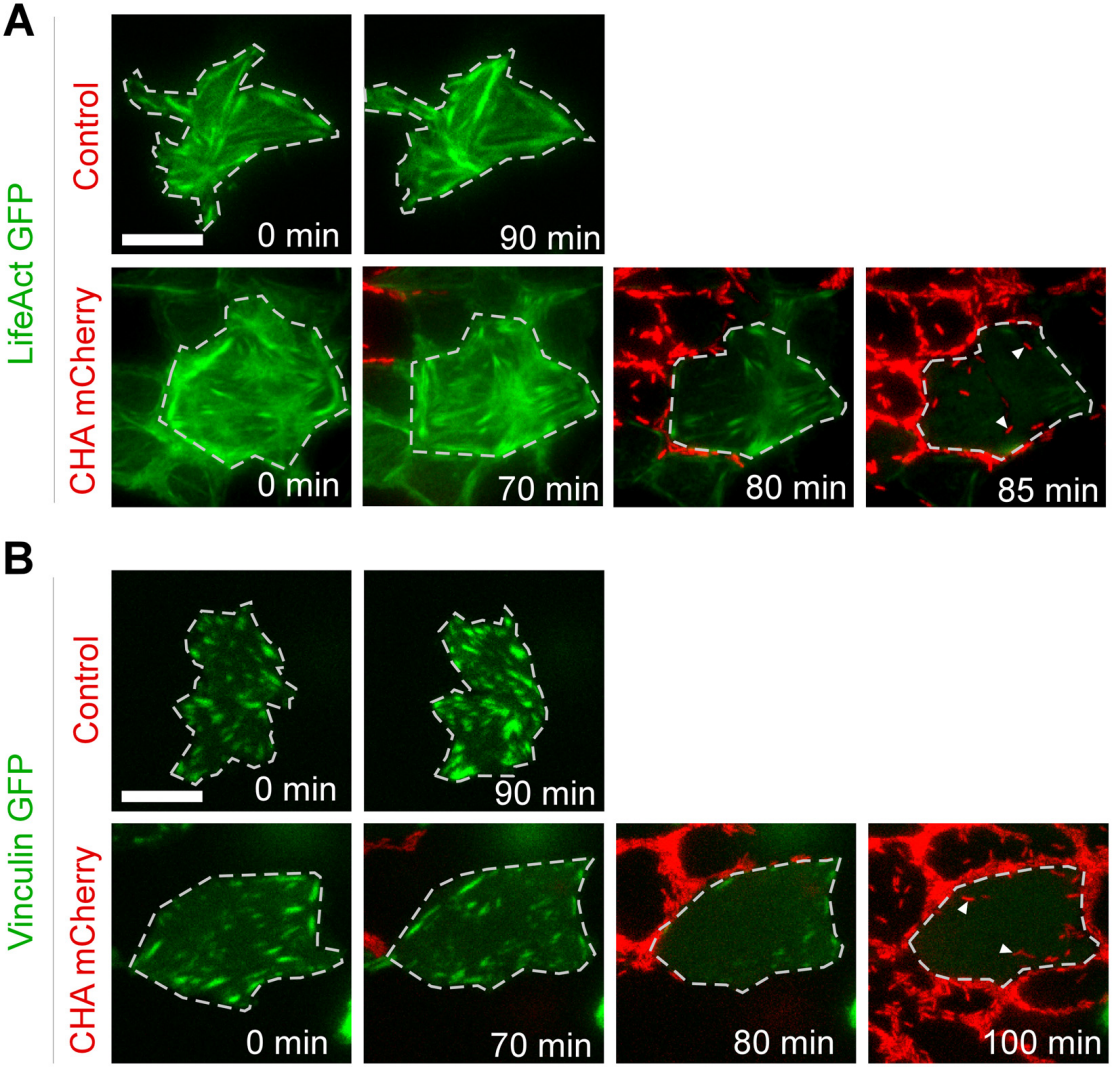
Correlation of bacteria propagation and disruption of host cellular components. (**A**) MDCK were transiently transfected with Lifeact-GFP to label actin fibers. Note that not all cells expressed Lifeact-GFP in the monolayer. Monolayers were infected with CHA-mCherry or uninfected (Control) and images were acquired in the basal compartment at different points by TIRF microscopy. Selected frames from S6 Movie are presented. For clarity, a transfected cell has been surrounded by a dashed line. Arrowheads highlight bacteria moving below the cell after actin fiber disruption. (**B**) Similar experiment but with vinculin-GFP expressing MDCK cells. Selected frames from S7 Movie.

To further examine the molecular events involved in bacterial propagation and how they correlate with toxin injection, we analyzed the biological effects of the toxins on the cytoskeleton and focal contacts in real-time. To do this, MDCK cells were transfected with either Lifeact-GFP—to label the actin cytoskeleton—or vinculin-GFP—to reveal focal contacts—and the basal compartment was observed by total internal reflectance fluorescence (TIRF) videomicroscopy. As previously reported [25, 26], toxin injection caused disruption of actin fibers and focal contacts (Fig 4A, 4B and S6 and S7 Movies). Bacteria only progressed below the cells after these two cellular components had been eliminated, suggesting that toxin injection took place when bacteria were still located at cell edges rather than directly under cells. Disruption of the cytoskeleton and focal contacts then facilitated bacterial propagation, as indicated by the arrowheads in Fig 4A and 4B.

Bacterial infiltration below the cells was also visible as a loss of fluorescence in the green channel (when cells were labeled with intracellular EGFP) at the basal side of the cell (S3 Fig), suggesting that bacteria raised the basal membrane, as previously hypothesized based on electron microscopy data [22]. Fluorescence was restored once the bacteria had moved away (S3 Fig), indicating that cells subsequently recovered their initial basal geometry, before cell rounding and detachment occurred.

### Bacterial migration under epithelial cells requires pili, T3SS and the flagellum

To analyze the effects of mutations of pili, the T3SS or the flagellum on bacterial migration under cells independently of transmigration across the epithelial layer, we generated an artificial wound in the MDCK layer prior to bacterial exposure and analyzed how bacteria progressed from this wound in real-time. With this cell system, mutants lacking pili never gained access to the basal compartment, while the T3SS and the flagellum mutants had severely reduced invasion capacities (S4 and S5A Figs). Thus, all three factors are required for motility below the cell layer. Interestingly, bacteria without a flagellum were present in the wound in much smaller numbers than the other strains (S4B Fig), suggesting an altered capacity to reach the wound. Even when present in the wound, the rate of invasion with this mutant was highly variable (S4B Fig), suggesting that it is a stochastic phenomenon. These observations hint that the flagellum plays an important role in migration to a point of weakness in the epithelial layer, and in its exploitation.

### Bacterial migration under the cells is related to twitching motility

We next investigated which characteristics of *P*. *aeruginosa* were necessary for migration under the epithelium. Pili-dependent twitching movement has been described as a type of collective migration used by *P*. *aeruginosa* under agar [31]. In our cell layers, bacteria initially migrated individually below epithelial cells, but were rapidly followed by cohorts of bacteria migrating together as rafts (S8 Movie). The leading bacterium progressed below the cells or under intercellular junctions, while the trailing bacteria within rafts mainly migrated below intercellular junctions.

**Fig 5:**
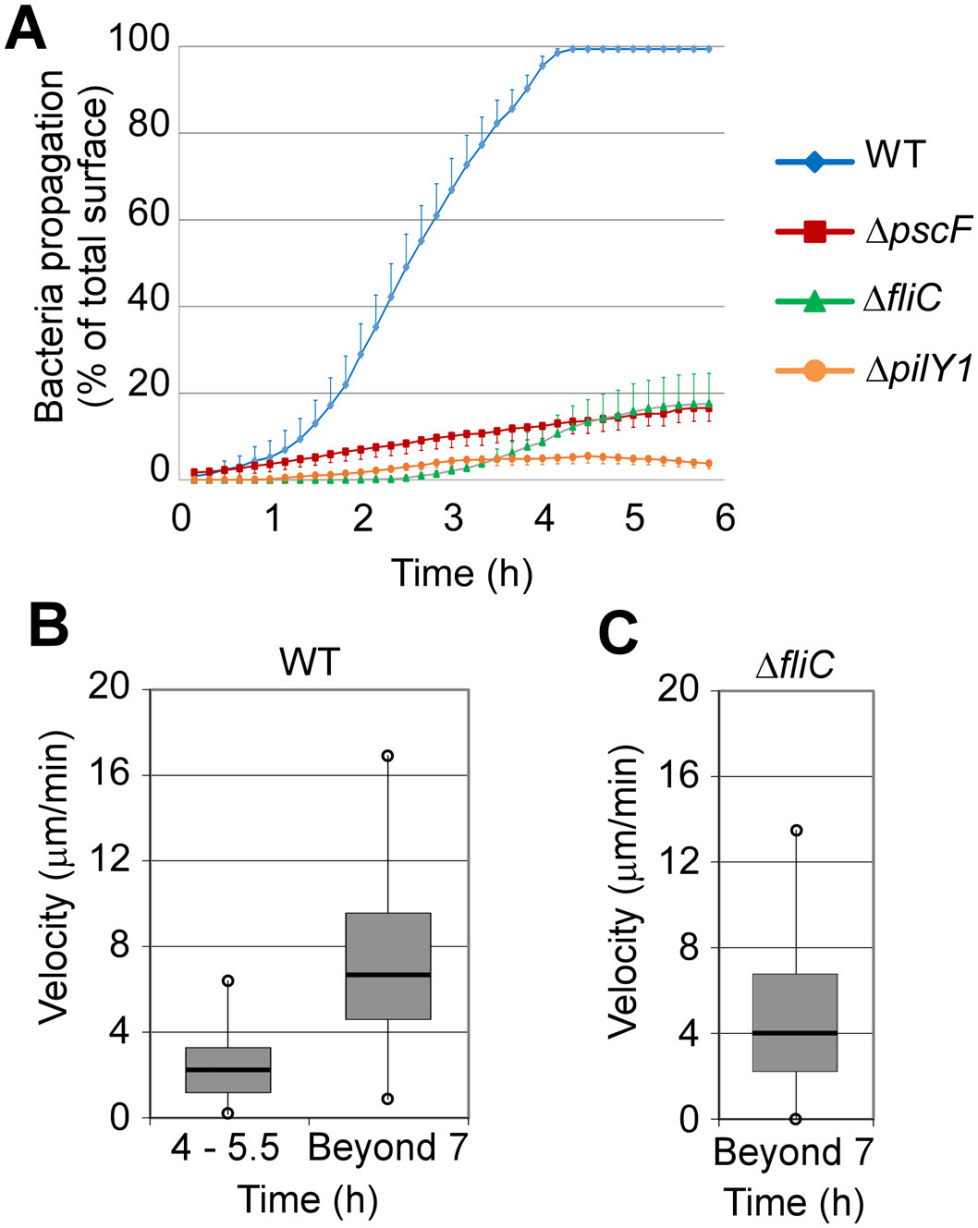
Bacterial propagation below an epithelial monolayer. (**A**) A wound was made in MDCK monolayers and bacteria were subsequently introduced in the medium. Invasion from the wound was recorded in the basal compartment by confocal microscopy at different time points, as illustrated in S4 Fig The wounded monolayers were infected with the wild type or with mutants lacking T3SS (Δ*pscF*), flagellum (Δ*fliC*) or pili (Δ*pilY1*). The invasion surfaces were measured in 8 fields. The data represent the averaged percentages of invaded surfaces (+ SEM) of total field surface minus the wounded area. (**B**) MDCK-EGFP monolayers were infected with CHA and bacteria progression was recorded by confocal microscopy in the basal compartment (S8 Movie). Bacteria were tracked using ImageJ software MTrackJ. Once bacteria assembled in rafts, the leading bacterium was recorded. The box-plots represent the instantaneous velocities recorded in 4 movies for each condition. N = 640 velocities from 40 trajectories at 4–5.5 hpi, and n = 455 velocities from 20 trajectories beyond 7 hpi. Data were significantly different at 4–5.5 and beyond 7 hpi, using Mann-Whitney's test (p < 0.001). (**C**) Similar experiment with Δ*fliC* (see S9 Movie). Box-plot showing the instantaneous velocities (n = 1360) of Δ*fliC* from 35 trajectories in 4 different movies.

The instantaneous velocities of bacteria were measured on several trajectories at the initial stages of bacterial migration (4–5.5 hpi) and at later time points (beyond 7 hpi) (Fig 5B). The median velocities were significantly different between these two time frames, progressing from 2.2 to 6.7 μm/min. These velocities are consistent with the median velocity reported for twitching under agar: ~ 3.5 μm/min [31]. Interestingly, the few flagellum-deficient bacteria that penetrated the basal compartment in transmigration experiments at late time points (7 hpi) (S9 Movie) had a comparable median velocity to wild-type cells (~4 μm/min) (Fig 5C), but only progressed under the cells as individual bacteria, suggesting that migration in rafts requires the flagellum.

These results indicate that the motility of *P*. *aeruginosa* beneath the epithelial cell layer involves a pili-dependent twitching mechanism, but collective behavior, including the formation of rafts, requires the flagellum.

## Discussion

Up to now, the precise mechanisms through which *P*. *aeruginosa* breaches the epithelial barrier have remained elusive. The fact that *P*. *aeruginosa* infection occurs in already established pathological settings suggests that specific biological events are required to facilitate bacterial dissemination. The results presented in this paper, based on a model epithelial barrier, provide new clues as to how *P*. *aeruginosa* transmigrates across an epithelium, and new information on the characteristics of its behavior in the basal compartment.

One of the major results of this study was the demonstration that *P*. *aeruginosa* uses a paracellular route to transmigrate, without requiring cellular internalization. In the tens of movies generated for this study, no bacterial internalization events were observed, thus eliminating a transcellular route. In previous studies [6, 7, 22], bacteria were observed in the basal compartment of infected epithelia in fixed samples, but no information on the route followed by the bacteria to reach this compartment was provided. Bacterial pathogens do not commonly employ a paracellular route across epithelia, a transcellular route being much more common. However, paracellular migration is described for specific examples, such as *Neisseria meningitidis* which uses it to cross the blood-brain barrier [32].

*P*. *aeruginosa* internalization by epithelial cells has been described in several studies, most of which used an antibiotic protection assay [33–35]. However, a recent report [36] suggests that the antibiotic protection assay may not be a relevant method to assess bacterial uptake by host cells as bacterial aggregates forming at the apical surface of cells are resistant to antibiotic treatment. Other groups reported electron microscopy images showing bacterial uptake in epithelial cells [37–39]. It is thus possible that cellular models other than MDCK or A549, or other infection conditions would be more appropriate to assess bacterial internalization.

In this article, we show for the first time that *P*. *aeruginosa* transmigration across MDCK layers takes place at sites of cell division and cell extrusion, but that bacteria exploit less than 5% of these occurrences. One explanation for this low level of exploitation might be statistical: there may be a low probability that patrolling bacteria would encounter cell-cell junctions in the apposite temporal window. Alternatively, perhaps not all division or extrusion sites are permissive to bacteria, and this process is controlled by other host factors (see below). In the time-frame of our experiments, the pathogen was not seen to specifically induce cell division or senescence, indicating that it is not a strategy initiated by *P*. *aeruginosa* to facilitate access to the sub-epithelium.

Although the entry site, once used by a leading bacterium, subsequently attracted many swimming bacteria, how bacteria are attracted so efficiently below the cells remains a major unsolved question and deserves further investigation. Conversely, the formation of immobile bacterial aggregates at the apical cell surface never triggered transmigration. Furthermore, bacterial aggregates did not specifically accumulate around transmigration sites. These two phenomena are therefore probably independent.

Interestingly, A549 monolayers, which establish immature cell-cell junctions, were highly permissive to bacterial penetration, indicating that adhesive proteins located at intercellular junctions constitute a main defense against bacterial dissemination. In agreement with these findings, E-cadherin, the major adhesive protein of adherens junctions promoting cell-cell interaction is not affected by the proteases secreted by *P*. *aeruginosa* [40–42]. In contrast, vascular endothelial (VE)-cadherin, expressed in vascular endothelia, is a substrate of *P*. *aeruginosa'*s elastase, LasB, making the vascular barrier vulnerable to invasion [41].

In this study, we observed that the interrupted E-cadherin junctions between dividing MDCK cells could be exploited by bacteria for transmigration. Imperfect sealing following division may be a rare phenomenon, which would explain why transmigration occurs at such a low rate. Alternatively, given that T3SS-deficient bacteria were unable to transmigrate, the bacterium may take advantage of exposed lateral membranes to inject T3SS toxins, eventually leading to loss of cell-cell contacts.

In cell monolayers, senescent cells are extruded apically. E-cadherin and tight junctions are lost by the apoptotic cells [43–46], but the neighboring cells rapidly move in to seal the gap. In this location, active junction remodeling takes place over a number of hours [29]. We observed that E-cadherin labeling was interrupted in the newly-formed junction, where bacterial penetration could occur. Further work will be needed to determine whether the gap exploited by bacteria for transmigration during cell division is pre-existing or triggered by the bacteria.

In systemic infections, *P*. *aeruginosa* mainly enters through the epithelium of lung alveoli. In normal situations, the alveolar epithelium is a slowly proliferating cell population, whereas in an injured epithelium the proliferation rate is considerably increased to allow for the replacement of injured cells [47, 48]. Thus, opportunities for mucosal penetration by bacteria may be available in these situations. However, the precise mechanisms of cell extrusion and cell division in this organ, particularly at the level of cell junctions, are unknown and constitute a major hurdle to our understanding of *P*. *aeruginosa* dissemination.

Bacterial transmigration across MDCK layers requires pili, the T3SS, and the flagellum; propagation below the monolayer involves the T3SS and pili, whereas the flagellum is only necessary for optimal invasion. The results presented here are the first to indicate involvement of the flagellum in *P*. *aeruginosa* transmigration across epithelial monolayers. As previously reported [49], like most Gram-negative pathogenic bacteria, *P*. *aeruginosa* does not inject T3SS toxins into the apical domain of epithelial cells. The cell's basolateral domain must therefore be accessible for pili-dependent bacterium-host cell contact to allow T3SS toxin injection [6, 18]. Therefore, it is not surprising that the T3SS and the pili mutants exhibited similar transmigration- and propagation-defective phenotypes.

Bacterial propagation under cells correlates with toxin injection and is followed by disruption of the cytoskeleton and focal contacts. The toxin effect may be a pre-requisite for bacterial propagation, as the absence of T3SS abolished invasion in the wound assay. Furthermore, bacteria only penetrated underneath the cells once actin fibers and focal contacts had been abolished, which can be easily explained by the need for the bacterium to lift the cell's basal membrane in order to progress. Interestingly, the flagellum mutants were only present in small numbers in wounds in the invasion assay, preventing efficient incursion into the basal compartment, while the other strains accumulated in this location whether or not they invaded the cell layer. The flagellum is thus needed to reach points where invasion opportunities are available. Although access of flagellum-deficient bacteria to the sub-basal compartment was delayed compared to the wild-type strain, once present, cells progressed with comparable speed, demonstrating that this motility parameter is independent of the flagellum.

This paper also reports the first characterization of *P*. *aeruginosa* migration under cell monolayers. In terms of speed and raft formation, the properties of wild-type bacterial migration under cells were similar to what has been described under agar [31], except that the leading bacteria migrated individually before assembling into rafts. Importantly, although the role of the flagellum in collective migration currently remains obscure, flagellum-deficient bacteria never formed rafts. It is thus possible that flagella may be required for collective migration under cells.

**Fig 6:**
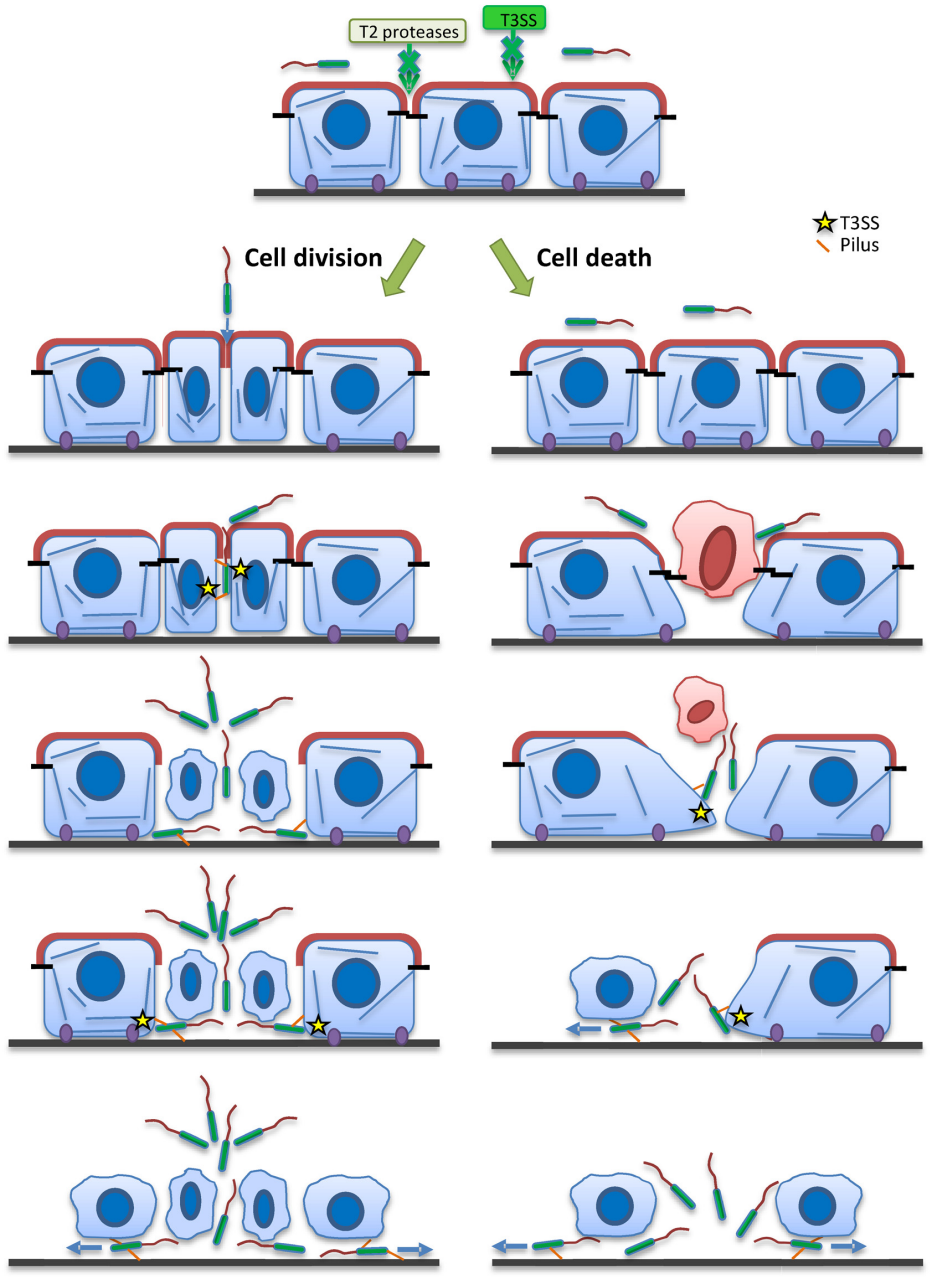
Model of *P*. *aeruginosa* transmigration across epithelial layers. In resting epithelium, T2SS proteases or T3SS toxins are inefficient to disrupt the barrier formed by polarized epithelial cells provided with mature junctions. Bacteria may use cell division or dying cell extrusion to penetrate the epithelium. Once bacteria have access to the baso-lateral domain, they interact with cell membranes using their pili and inject their T3SS toxins, inducing cell retraction and facilitating their invasion of the basal compartment.

Based on the main data presented in this paper, we propose a model (Fig 6) in which bacteria take advantage of either cell division sites or sites of senescent cell extrusion to transmigrate across an epithelial layer. At these specific sites, junctional sealing is imperfect or the basolateral membranes are partially exposed allowing toxin injection. Transmigration of the first cell is rapidly followed by massive bacterial infiltration and access to the basal compartment. Once beneath the cell layer, the T3SS toxins induce cell rounding and detachment from the extracellular matrix, promoting rapid bacterial propagation below cells, rapidly undermining the epithelium.

In conclusion, this paper describes a chronological view of *P*. *aeruginosa* transmigration across a model epithelium. The bacteria exploit rare, specific characteristics of the monolayer to breach the barrier at intercellular junctions and to propagate underneath it, employing at least three virulence factors: the T3SS system, pili and the flagellum.

## Materials and Methods

### Genetic constructions

Replicative pIApX2mCherry plasmid derived from pIApX2 (32) by replacing the *gfp* reporter gene fused to constitutive and strong *pX2* promoter by mCherry coding sequence. The *pX2-mCherry* fusion was subcloned into mini-CTX integrative plasmid leading to miniCTXpX2mCherry plasmid.

### *P*. *aeruginosa* strains and culture

The strains and the plasmids used in this study are described in S1 Table. The bacteria of CHA strain secrete ExoS, T and Y through their T3SS, and exhibit functional pili and flagellum [50–52]. CHAΔ*pilY1* and CHAΔ*fliC* were checked for defective twitching and swimming, respectively (S5 Fig) and CHAΔ*pscF* was previously shown to have impaired T3SS toxin secretion [52]. All strains were transformed with pIAPX2-mCherry plasmid for fluorescence imaging. Bacteria were grown in liquid LB medium at 37°C with agitation until the cultures reached an optical density value of 1.0 to be in the exponential growth phase.

### Cell culture and infection

A549-EGFP (Enhanced Green Fluorescent Protein)(this study) and MDCK-EGFP (a gift from René-Marc Mège) cells were grown in RPMI and DMEM medium, respectively, supplemented with 10% fetal calf serum (all from Invitrogen). For videomicroscopy experiments, cells were seeded at 2,500 cells per mm^2^ on Labtek II 8-chambered (Thermo Fisher Scientific) coverslips and used 72 h later to obtain highly confluent monolayers. Medium was replaced 16 h before infection with fresh medium, and 1 h before infection with fresh non-supplemented medium. Cells were infected at a multiplicty of infection (MOI) of 15 (unless otherwise indicated) and immediately observed by videomicroscopy.

### Cell transfection

The plasmid coding for Lifeact-EGFP (Fig 4A) was obtained from Roland Wedlich-Soldner. The vinculin-GFP plasmid (Fig 4B) was from Hélène Delanoë-Ayari. MDCK cells were transfected with expression plasmids using Lipofectamin 2000 (LifeTechnologies) 48 h prior to infection. The medium was replaced 24 h before infection with fresh medium.

### Visualization of bacterial transmigration and cell retraction during infection

A549-EGFP and MDCK-EGFP cells were grown as described above, and were infected by CHA-mCherry. Bacterial transmigration was observed by confocal microscopy for 6 hours, with time points every 15 min. Successive planes in 3D stacks were taken every 0.4 μm. Out-of-focus information in confocal images was removed using the restoration module from Volocity (PerkinElmer). Iterative deconvolution was performed with calculated point spread function. 3D-recontructions of confocal time-lapse were performed using the 3D VTK module from Icy software.

For quantitative analysis of bacteria transmigration (Fig 1C and 1D) and cell retraction (Fig 1E and 1F), 4 different mCherry transfected-strains were filmed in parallel. For each strain, 10 randomly chosen positions were imaged as above. The infection was filmed for 7.5 h, with time points every 15 min. To minimize the acquisition time, only the basal part of the cells was imaged (11 z-planes). For bacteria counting, only the z-sections where transmigrated bacteria were on focus were used. Images were binarized and bacteria were counted using ImageJ software. For cell retraction quantification, projections of the 5 lowest z-sections of A549-EGFP or MDCK-EGFP confocal images were generated. The projections were then binarized and the total cell surfaces were calculated for each image.

### Quantification of cell division and cell death

MDCK-EGFP were grown as described above. One hour prior to experiment, medium was replaced with fresh non-supplemented medium and nuclei were labeled with Hoechst 33342 (1 μg/mL final concentration, Sigma-Aldrich). Nuclei and bacteria were filmed every 15 min with a confocal microscope and a 20X air objective. 48 fields were filmed in parallel, at 16 z-planes separated by 1 μm. Nuclei were counted on the first time point using ImageJ. Transmigration sites were scored visually, and classified according to their properties (i.e. cell division or cell death) (Fig 2B). Cell divisions and cell deaths were scored visually for the first 3 hours of infection (Fig 2C). Comparison of cell division numbers in infected and non-infected conditions was performed in another set of experiment, in which 24 infected fields and 24 non-infected fields were imaged in parallel. Cell division and cell death were scored visually for the first 3 hours of infection (Fig 2D and 2E).

### Visualization of E-cadherin labeling at transmigration sites

MDCK Ecad-GFP were grown as described for MDCK-EGFP. One hour prior to experiment, medium was replaced with fresh non-supplemented medium and nuclei were labeled with Hoechst 33342. Then, cells were infected by CHA-mCherry. Bacterial transmigration was observed by confocal microscopy for 6 hours, with time points every 15 min, at 40 z-planes separated by 0.4 μm.

### Correlation between bacteria transmigration and toxin injection

Protocol for CCF2/AM loading was adapted from previous studies to be used in real-time microscopy [30]. Briefly, after 3 h of infection with CHA Δ*exoS-Sbla*-mCherry, cells were washed with PBS containing 2.5 mM probenicid, and incubated with freshly prepared CCF2-AM solution (2 μM final concentration, LiveBLAzer FRET—B/G Loading Kit, Life Technologies) for 90 min in the dark at room temperature. Then, cells were carefully washed twice with PBS-2.5 mM probenicid. Fluorescence images were acquired with a wide-field microscope as described below. Images of bacteria were also captured, either on the basal (i.e. transmigrated bacteria) or on the apical side of MDCK. To distinguish between these two populations of bacteria in merged images, transmigrated bacteria were given a red artificial color, whereas bacteria on the apical side were colored in yellow (Fig 3C).

To determine whether bacteria in basal compartment are located below injected or uninjected cells, 12 fields exhibiting injected cells were acquired by wide-field microscopy. For each field, the red channel (bacteria) was binarized, and the total number of transmigrated bacteria was measured using ImageJ. On the same images, the blue channel (cleaved CCF2) was binarized, and a mask corresponding to the zone of injected cells was generated. The number of bacteria in the mask was calculated. As the CCF2 fluorescence shift is delayed compared to bacterial propagation under cells, the number of bacteria surrounding the blue cells was also measured in the same mask dilated of 15 μm (Fig 3D).

### Measurement of bacterial propagation from a wound in the epithelial monolayer

Cells were grown as indicated above. One hour prior to infection, the monolayer was scratched with a sterile 10-μL pipette tip across the well median. The well was then washed twice with fresh non-supplemented medium.

Wounded MDCK-EGFP monolayers were infected with 4 mCherry transfected-strains in parallel (S4 Fig). For each strain, 8 positions were imaged by confocal microscopy. The infection was filmed for 6 hours, with time points every 10 min. Only the basal part of the cells was imaged (11 z-planes separated by 0.4 μm). The surface occupied by bacteria under MDCK was measured using ImageJ. Briefly, for each time point, a polygon representing the area invaded by bacteria under the cells was manually drawn. Its surface was then measured and compared to the total cell surface minus the wounded area (Fig 5A).

### Tracking of transmigrated bacteria

MDCK-EGFP were infected with CHA-mCherry. After 4 h of infection, early transmigration sites were localized visually on a confocal microscope and bacteria propagation from these sites was filmed for 1.5 h every 30 s (S8 and S9 Movies). Tracking of bacteria at the propagation leading edge was performed using the MTrackJ plugin of ImageJ. The instantaneous velocities were measured (Fig 5B and 5C). Only tracks of more than 20 μm were considered.

### Confocal microscopy

For fluorescence time-lapse microscopy, cells were imaged using a confocal spinning-disk inverted microscope (Nikon TI-E Eclipse) equipped with an Evolve EMCCD camera. The optical sectioning was performed by a Yokogawa motorized confocal head CSUX1-A1. Images were acquired using an illumination system from Roper Scientific (iLasPulsed) with a CFI Plan APO VC oil immersion objective (60X, N.A 1.4) or CFI Plan Fluor oil immersion objective (40X, N.A. 1.3). Z-series were generated using a motorized Z-piezo stage (ASI) by acquiring images with a step size of 0.4 μm. Microscope was controlled with MetaMorph software (Molecular Devices). Temperature, CO_2_, and humidity control was performed using a chamlide TC system (TC-A, Quorum technologies). Solid-state 405, 491 and 561 nm lasers (iLas, Roper Scientific) and ET 460/50M (Chroma), ET 525/50M (Chroma), FF01-605/54 (Semrock) emission filters were used for excitation and emission of Hoechst 33342, EGFP and mCherry fluorescence, respectively.

### Wide-field videomicroscopy

Labtek chambers containing the infected cells were placed in an incubator equilibrated at 37°C and 5% CO_2_ located on a IX71 Olympus microscope controlled by the CellR Olympus system, automated in x, y, z axis and driven by Xcellence software (Olympus). Uncleaved CCF2 (i.e. non-injected cells) was excited at 360/40 nm, and emission was collected at 535/50 nm. Cleaved CCF2 (i.e. injected cells) were excited at 360/40 nm, and emission was collected at 460/50 nm. Images were captured with a Hammamatsu Orca-ER camera and a 20X (N.A. 0.5) air objective (UPLFN20XPH, Olympus). Acquisition times and frequencies are indicated in the figure legend.

### Visualization of cell intoxication and bacterial propagation by TIRF microscopy

For TIRF microscopy, Lifeact or vinculin-transfected cells were imaged using a NIKON TI-E/B microscope, equipped with an Evolve EMCCD camera. The illumination system used was from from Roper Scientific (iLas^2^), and the objective was a Nikon CFI Plan APO VC oil immersion objective (60X, N.A. 1.4). The microscope was controlled with MetaMorph software (Molecular Devices). Temperature, CO_2_, and humidity control was performed using a chamlide TC system (TC-A, Quorum technologies). The laser used for excitation of EGFP was a 491 nm cobolt Calypso laser, and the one for excitation of mCHerry was a 561 nm MPBC Green Visible Fiber laser. ET525/50M (Chroma) and ET605/52M (Chroma) emission filters were used for for EGFP and mCherry fluorescence collection, respectively.

### Immunolabelling

MDCK and A549 cells were washed, fixed with formaldehyde, permeabilized with 0.5% triton and labeled with a rabbit anti-ZO1 primary antibody (Zymed) and goat anti-rabbit A488 secondary antibody (Molecular Probes). Nuclei were labeled with Hoechst 33258 (10 μg/mL, Sigma-Aldrich). Preparations were observed with an Olympus inverted microscope with a 20x air objective.

### Motility assays

For twitching motility, bacteria were inoculated at the plastic-agar interface (10 g/L tryptone, 5 g/L yeast extract, 1% agar, 10 g/L NaCl). After 48 h at 37°C, the agar medium was removed, and the twitching zone was observed after coomassie blue staining.

Bacterial swimming was observed after 48 h at 30°C in 0.3% agar medium plate (10 g/L tryptone, 5 g/L NaCl)(S5 Fig).

### Statistics

All experiments shown here are representative of at least 3 independent experiments. Mann-Whitney's test was performed using StatXact software.

## Supporting Information

S1 Table Strains and plasmids used in this work. (DOCX)

S1 Fig E-cadherin labeling of MDCK and A549 monolayers. MDCK (**A**) or A549 (**B**) cells, seeded at 2,500 cells/mm^2^ and cultured for 3 days, as for videomicroscopy experiments, were fixed and labeled with anti-ZO-1 antibodies (green) to highlight cell-cell junctions. Nuclei are in blue. Yellow arrowheads show ZO-1-positive junctions and white arrowheads show interrupted junctions. Scale bars: 50 μm.
(PDF)

S2 Fig Kinetics of bacteria transmigration across MDCK monolayers. (**A,B**) MDCK-EGFP monolayers were infected with various strains expressing mCherry. Merged confocal images of green and red channels, taken at the cell basal side at different time points are shown. Note that MDCK cells did not express EGFP at the same level, allowing cell individualization. (**A**) MDCK were infected by CHA or with CHA mutants lacking T3SS (ΔpscF), pili (ΔpilY1) or flagellum (ΔfliC). Note that at late time points, cells infected with ΔpscF or ΔfliC exhibited disrupted intercellular junctions (arrowheads), a phenotype not observed in ΔpilY1-infected cells. Quantifications of bacterial transmigration and cell retraction are shown in Fig 1C and 1E. (**B**) Similar experiment using PAK background. MDCK were infected with PAK. Scale bars, 20 μm.
(PDF)

S3 Fig Bacteria raise the host cells to invade the lower compartment. MDCK-EGFP cells were infected with CHA-mCherry. Two successive confocal microscopy images captured at the cell’s basal side were selected to show the imprints of bacteria (arrowheads) in the cell, as monitored by the loss of green fluorescence in MDCK cytosol. Scale bars: 15 μm.
(PDF)

S4 Fig Bacterial propagation below an epithelial monolayer from a wound. A wound was made in MDCK monolayers and bacteria were subsequently introduced in the medium. Invasion from the wound was recorded in the basal compartment by confocal microscopy at different time points, as indicated. The green lines indicate the wound edge. Scale bars: 25 μm. (**A**) The wounded monolayer was infected by CHA. (**B**) The wounded monolayers were infected with mutants lacking T3SS (ΔpscF), flagellum (ΔfliC) or pili (ΔpilY1). Note that in the ΔfliC condition, bacteria did not accumulate in the wounded area and invaded solely from specific points of the wound (“Invasion” as opposed to “No invasion”). See Fig 5 for quantifications.
(PDF)

S5 Fig Swimming and twitching motility behavior of CHAΔ*pscF*, CHAΔ*fliC* and CHAΔ*pilY1*. The motion types were assessed using standard procedures for the 3 mutants, compared to wild type.
(TIF)

S1 Movie 3D-reconstruction of CHA transmigration across MDCK cell layer. The images illustrate the invasion of MDCK monolayer (green) by bacteria (red), viewed from the top or the bottom side. See Fig 1A for more details.
(AVI)

S2 Movie 3D-reconstruction of CHA transmigration across A549 cell layer. The images illustrate the invasion of A549 monolayer (green) by bacteria (red), viewed from the bottom side. See Fig 1B for more details.
(AVI)

S3 Movie Penetration of a bacterium at a cell division site. E-cadherin-GFP-MDCK cells exhibiting a gap in E-cadherin labeling between two dividing cells. A bacterium (red) penetrates the layer at this specific point. See Fig 2G for more details.
(AVI)

S4 Movie Penetration of bacteria at senescent cell extrusion site. E-cadherin-GFP-MDCK cells exhibiting a gap in E-cadherin labeling in the junction newly formed below the extruding dying cell. Several bacteria (red) penetrate the layer at this specific point. See Fig 2H for more details.
(AVI)

S5 Movie T3SS toxin injection is correlated with bacterial progression under the cells. T3SS toxin injection was monitored using the ExoS-Bla/CCF2 system (See Fig 3C for more details). Uninjected cells are green, injected cells are blue and the bacteria located in the basal compartment are red.
(AVI)

S6 Movie Disruption of actin cytoskeleton in the course of bacterial progression under the cells. The actin cytoskeleton of MDCK was labeled with Lifeact-GFP. Left: actin skeleton, middle: bacteria, right: merge images. The bacteria penetrated the basal compartment when the cytoskeleton vanished, owing to T3SS toxin injection. See Fig 4A for more details.
(AVI)

S7 Movie Disruption of focal contacts in the course of bacterial progression under the cells. MDCK focal contacts were labeled with vinculin-GFP. Left: focal contacts, middle: bacteria, right: merge. The bacteria penetrated the basal compartment when the focal contacts vanished, owing to T3SS toxin injection. See Fig 4B for more details.
(AVI)

S8 Movie Tracking of wild-type bacteria in the basal compartment. Some trajectories (colorized) of wild-type bacteria (white) invading the basal compartment were followed using the MTrackJ plugin of ImageJ software. See Fig 5B for quantifications.
(AVI)

S9 Movie Tracking of flagellum-deficient bacteria in the basal compartment. Some trajectories (colorized) of Δ*fliC* bacteria (white) invading the basal compartment were followed using the MTrackJ plugin of ImageJ software. See Fig 5C for quantifications.
(AVI)

## References

[1] Gellatly SL, Hancock RE. Pseudomonas aeruginosa: new insights into pathogenesis and host defenses. Pathogens and disease. 2013;67(3):159–73. 10.1111/2049-632X.12033 . 23620179

[2] Ramphal R, Small PM, Shands JW Jr., Fischlschweiger W, Small PA Jr. Adherence of Pseudomonas aeruginosa to tracheal cells injured by influenza infection or by endotracheal intubation. Infection and immunity. 1980;27(2):614–9. 6769805 10.1128/iai.27.2.614-619.1980 PMC550808

[3] de Bentzmann S, Roger P, Puchelle E. Pseudomonas aeruginosa adherence to remodelling respiratory epithelium. The European respiratory journal. 1996;9(10):2145–50. . 8902481 10.1183/09031936.96.09102145

[4] Fleiszig SM, Evans DJ, Do N, Vallas V, Shin S, Mostov KE. Epithelial cell polarity affects susceptibility to Pseudomonas aeruginosa invasion and cytotoxicity. Infection and immunity. 1997;65(7):2861–7. 9199460 10.1128/iai.65.7.2861-2867.1997 PMC175402

[5] Lee A, Chow D, Haus B, Tseng W, Evans D, Fleiszig S, et al Airway epithelial tight junctions and binding and cytotoxicity of Pseudomonas aeruginosa. The American journal of physiology. 1999;277(1 Pt 1):L204–17. . 10409249 10.1152/ajplung.1999.277.1.L204

[6] Heiniger RW, Winther-Larsen HC, Pickles RJ, Koomey M, Wolfgang MC. Infection of human mucosal tissue by Pseudomonas aeruginosa requires sequential and mutually dependent virulence factors and a novel pilus-associated adhesin. Cellular microbiology. 2010;12(8):1158–73. 10.1111/j.1462-5822.2010.01461.x 20331639 PMC2906647

[7] Soong G, Parker D, Magargee M, Prince AS. The type III toxins of Pseudomonas aeruginosa disrupt epithelial barrier function. Journal of bacteriology. 2008;190(8):2814–21. 10.1128/JB.01567-07 18165298 PMC2293221

[8] Deng Q, Barbieri JT. Molecular mechanisms of the cytotoxicity of ADP-ribosylating toxins. Annual review of microbiology. 2008;62:271–88. 10.1146/annurev.micro.62.081307.162848 18785839

[9] Hauser AR. The type III secretion system of Pseudomonas aeruginosa: infection by injection. Nature reviews. 2009;7(9):654–65. 10.1038/nrmicro2199 19680249 PMC2766515

[10] El-Solh AA, Hattemer A, Hauser AR, Alhajhusain A, Vora H. Clinical outcomes of type III Pseudomonas aeruginosa bacteremia. Critical care medicine. 2012;40(4):1157–63. 10.1097/CCM.0b013e3182377906 22080633 PMC3288436

[11] Hauser AR, Cobb E, Bodi M, Mariscal D, Valles J, Engel JN, et al Type III protein secretion is associated with poor clinical outcomes in patients with ventilator-associated pneumonia caused by Pseudomonas aeruginosa. Critical care medicine. 2002;30(3):521–8. . 11990909 10.1097/00003246-200203000-00005

[12] Le Berre R, Nguyen S, Nowak E, Kipnis E, Pierre M, Quenee L, et al Relative contribution of three main virulence factors in Pseudomonas aeruginosa pneumonia. Critical care medicine. 2011;39(9):2113–20. 10.1097/CCM.0b013e31821e899f . 21572326

[13] Vance RE, Rietsch A, Mekalanos JJ. Role of the type III secreted exoenzymes S, T, and Y in systemic spread of Pseudomonas aeruginosa PAO1 in vivo. Infection and immunity. 2005;73(3):1706–13. . 15731071 10.1128/IAI.73.3.1706-1713.2005 PMC1064930

[14] Rangel SM, Diaz MH, Knoten CA, Zhang A, Hauser AR. The Role of ExoS in Dissemination of Pseudomonas aeruginosa during Pneumonia. PLoS pathogens. 2015;11(6):e1004945 10.1371/journal.ppat.1004945 26090668 PMC4474835

[15] Murray TS, Ledizet M, Kazmierczak BI. Swarming motility, secretion of type 3 effectors and biofilm formation phenotypes exhibited within a large cohort of Pseudomonas aeruginosa clinical isolates. Journal of medical microbiology. 2010;59(Pt 5):511–20. 20093376 10.1099/jmm.0.017715-0 PMC2855384

[16] Feldman M, Bryan R, Rajan S, Scheffler L, Brunnert S, Tang H, et al Role of flagella in pathogenesis of Pseudomonas aeruginosa pulmonary infection. Infection and immunity. 1998;66(1):43–51. 9423837 10.1128/iai.66.1.43-51.1998 PMC107856

[17] Mattick JS. Type IV pili and twitching motility. Annual review of microbiology. 2002;56:289–314. 10.1146/annurev.micro.56.012302.160938 . 12142488

[18] Hayashi N, Nishizawa H, Kitao S, Deguchi S, Nakamura T, Fujimoto A, et al Pseudomonas aeruginosa injects type III effector ExoS into epithelial cells through the function of type IV pili. FEBS letters. 2015;589(8):890–6. 10.1016/j.febslet.2015.02.031 . 25747138

[19] Bucior I, Pielage JF, Engel JN. Pseudomonas aeruginosa pili and flagella mediate distinct binding and signaling events at the apical and basolateral surface of airway epithelium. PLoS pathogens. 2012;8(4):e1002616 10.1371/journal.ppat.1002616 22496644 PMC3320588

[20] Kierbel A, Gassama-Diagne A, Rocha C, Radoshevich L, Olson J, Mostov K, et al Pseudomonas aeruginosa exploits a PIP3-dependent pathway to transform apical into basolateral membrane. The Journal of cell biology. 2007;177(1):21–7. . 17403925 10.1083/jcb.200605142 PMC2064102

[21] Kierbel A, Gassama-Diagne A, Mostov K, Engel JN. The phosphoinositol-3-kinase-protein kinase B/Akt pathway is critical for Pseudomonas aeruginosa strain PAK internalization. Molecular biology of the cell. 2005;16(5):2577–85. . 15772151 10.1091/mbc.E04-08-0717 PMC1087259

[22] Apodaca G, Bomsel M, Lindstedt R, Engel J, Frank D, Mostov KE, et al Characterization of Pseudomonas aeruginosa-induced MDCK cell injury: glycosylation-defective host cells are resistant to bacterial killing. Infection and immunity. 1995;63(4):1541–51. 7890421 10.1128/iai.63.4.1541-1551.1995 PMC173187

[23] Bucior I, Mostov K, Engel JN. Pseudomonas aeruginosa-mediated damage requires distinct receptors at the apical and basolateral surfaces of the polarized epithelium. Infection and immunity. 2010;78(3):939–53. 10.1128/IAI.01215-09 20008530 PMC2825949

[24] Dacheux D, Goure J, Chabert J, Usson Y, Attree I. Pore-forming activity of type III system-secreted proteins leads to oncosis of Pseudomonas aeruginosa-infected macrophages. Molecular microbiology. 2001;40(1):76–85. . 11298277 10.1046/j.1365-2958.2001.02368.x

[25] Fraylick JE, La Rocque JR, Vincent TS, Olson JC. Independent and coordinate effects of ADP-ribosyltransferase and GTPase-activating activities of exoenzyme S on HT-29 epithelial cell function. Infection and immunity. 2001;69(9):5318–28. . 11500401 10.1128/IAI.69.9.5318-5328.2001 PMC98641

[26] Huber P, Bouillot S, Elsen S, Attree I. Sequential inactivation of Rho GTPases and Lim kinase by Pseudomonas aeruginosa toxins ExoS and ExoT leads to endothelial monolayer breakdown. Cell Mol Life Sci. 2014;71(10):1927–41. 10.1007/s00018-013-1451-9 . 23974244 PMC11113219

[27] Kazmierczak BI, Engel JN. Pseudomonas aeruginosa ExoT acts in vivo as a GTPase-activating protein for RhoA, Rac1, and Cdc42. Infection and immunity. 2002;70(4):2198–205. . 11895987 10.1128/IAI.70.4.2198-2205.2002 PMC127837

[28] Krall R, Schmidt G, Aktories K, Barbieri JT. Pseudomonas aeruginosa ExoT is a Rho GTPase-activating protein. Infection and immunity. 2000;68(10):6066–8. . 10992524 10.1128/iai.68.10.6066-6068.2000 PMC101576

[29] Pentecost M, Otto G, Theriot JA, Amieva MR. Listeria monocytogenes invades the epithelial junctions at sites of cell extrusion. PLoS pathogens. 2006;2(1):e3 10.1371/journal.ppat.0020003 16446782 PMC1354196

[30] Verove J, Bernarde C, Bohn YS, Boulay F, Rabiet MJ, Attree I, et al Injection of Pseudomonas aeruginosa Exo toxins into host cells can be modulated by host factors at the level of translocon assembly and/or activity. PloS one. 2012;7(1):e30488 10.1371/journal.pone.0030488 22299042 PMC3267729

[31] Gloag ES, Turnbull L, Huang A, Vallotton P, Wang H, Nolan LM, et al Self-organization of bacterial biofilms is facilitated by extracellular DNA. Proceedings of the National Academy of Sciences of the United States of America. 2013;110(28):11541–6. 10.1073/pnas.1218898110 23798445 PMC3710876

[32] Join-Lambert O, Morand PC, Carbonnelle E, Coureuil M, Bille E, Bourdoulous S, et al Mechanisms of meningeal invasion by a bacterial extracellular pathogen, the example of Neisseria meningitidis. Progress in neurobiology. 2010;91(2):130–9. 10.1016/j.pneurobio.2009.12.004 . 20026234

[33] Kwong MS, Evans DJ, Ni M, Cowell BA, Fleiszig SM. Human tear fluid protects against Pseudomonas aeruginosa keratitis in a murine experimental model. Infection and immunity. 2007;75(5):2325–32. 10.1128/IAI.01404-06 17325054 PMC1865794

[34] Sana TG, Hachani A, Bucior I, Soscia C, Garvis S, Termine E, et al The second type VI secretion system of Pseudomonas aeruginosa strain PAO1 is regulated by quorum sensing and Fur and modulates internalization in epithelial cells. The Journal of biological chemistry. 2012;287(32):27095–105. 10.1074/jbc.M112.376368 22665491 PMC3411052

[35] Schaible B, McClean S, Selfridge A, Broquet A, Asehnoune K, Taylor CT, et al Hypoxia modulates infection of epithelial cells by Pseudomonas aeruginosa. PloS one. 2013;8(2):e56491 10.1371/journal.pone.0056491 23418576 PMC3572047

[36] Tran CS, Rangel SM, Almblad H, Kierbel A, Givskov M, Tolker-Nielsen T, et al The Pseudomonas aeruginosa type III translocon is required for biofilm formation at the epithelial barrier. PLoS pathogens. 2014;10(11):e1004479 10.1371/journal.ppat.1004479 25375398 PMC4223071

[37] Chi E, Mehl T, Nunn D, Lory S. Interaction of Pseudomonas aeruginosa with A549 pneumocyte cells. Infection and immunity. 1991;59(3):822–8. 1671777 10.1128/iai.59.3.822-828.1991 PMC258333

[38] Emam A, Carter WG, Lingwood C. Glycolipid-Dependent, Protease Sensitive Internalization of Pseudomonas aeruginosa Into Cultured Human Respiratory Epithelial Cells. The open microbiology journal. 2010;4:106–15. 10.2174/1874285801004010106 21270937 PMC3026333

[39] Fleiszig SM, Zaidi TS, Fletcher EL, Preston MJ, Pier GB. Pseudomonas aeruginosa invades corneal epithelial cells during experimental infection. Infection and immunity. 1994;62(8):3485–93. 8039920 10.1128/iai.62.8.3485-3493.1994 PMC302982

[40] Clark CA, Thomas LK, Azghani AO. Inhibition of protein kinase C attenuates Pseudomonas aeruginosa elastase-induced epithelial barrier disruption. American journal of respiratory cell and molecular biology. 2011;45(6):1263–71. 10.1165/rcmb.2010-0459OC . 21757681

[41] Golovkine G, Faudry E, Bouillot S, Voulhoux R, Attree I, Huber P. VE-cadherin cleavage by LasB protease from Pseudomonas aeruginosa facilitates type III secretion system toxicity in endothelial cells. PLoS pathogens. 2014;10(3):e1003939 10.1371/journal.ppat.1003939 24626230 PMC3953407

[42] Nomura K, Obata K, Keira T, Miyata R, Hirakawa S, Takano K, et al Pseudomonas aeruginosa elastase causes transient disruption of tight junctions and downregulation of PAR-2 in human nasal epithelial cells. Respiratory research. 2014;15:21 10.1186/1465-9921-15-21 24548792 PMC3936699

[43] Bojarski C, Weiske J, Schoneberg T, Schroder W, Mankertz J, Schulzke JD, et al The specific fates of tight junction proteins in apoptotic epithelial cells. Journal of cell science. 2004;117(Pt 10):2097–107. 10.1242/jcs.01071 . 15054114

[44] Keller SH, Nigam SK. Biochemical processing of E-cadherin under cellular stress. Biochemical and biophysical research communications. 2003;307(2):215–23. . 12859942 10.1016/s0006-291x(03)01143-4

[45] Schmeiser K, Grand RJ. The fate of E- and P-cadherin during the early stages of apoptosis. Cell death and differentiation. 1999;6(4):377–86. 10.1038/sj.cdd.4400504 . 10381631

[46] Steinhusen U, Weiske J, Badock V, Tauber R, Bommert K, Huber O. Cleavage and shedding of E-cadherin after induction of apoptosis. The Journal of biological chemistry. 2001;276(7):4972–80. 10.1074/jbc.M006102200 . 11076937

[47] Guillot L, Nathan N, Tabary O, Thouvenin G, Le Rouzic P, Corvol H, et al Alveolar epithelial cells: master regulators of lung homeostasis. The international journal of biochemistry & cell biology. 2013;45(11):2568–73. 10.1016/j.biocel.2013.08.009 . 23988571

[48] Uhal BD. Cell cycle kinetics in the alveolar epithelium. The American journal of physiology. 1997;272(6 Pt 1):L1031–45. . 9227501 10.1152/ajplung.1997.272.6.L1031

[49] Doran KS, Banerjee A, Disson O, Lecuit M. Concepts and mechanisms: crossing host barriers. Cold Spring Harb Perspect Med. 2013;3:a010090 10.1101/cshperspect.a010090 23818514 PMC3685877

[50] Dacheux D. Mise en évidence et étude de la cytotoxicité de Pseudomonas aeruginosa envers les neutrophiles humains. [PhD thesis]2000.

[51] Dacheux D, Attree I, Schneider C, Toussaint B. Cell death of human polymorphonuclear neutrophils induced by a Pseudomonas aeruginosa cystic fibrosis isolate requires a functional type III secretion system. Infection and immunity. 1999;67(11):6164–7. 10531282 10.1128/iai.67.11.6164-6167.1999 PMC97008

[52] Pastor A, Chabert J, Louwagie M, Garin J, Attree I. PscF is a major component of the Pseudomonas aeruginosa type III secretion needle. FEMS microbiology letters. 2005;253(1):95–101. . 16239085 10.1016/j.femsle.2005.09.028

