# Supplementary material for: Pseudomonas aeruginosa Transmigrates at Epithelial Cell-Cell Junctions, Exploiting Sites of Cell Division and Senescent Cell Extrusion
Source: PLoS Pathog. 2016 Jan 4;12(1):e1005377. doi: 10.1371/journal.ppat.1005377 (PMC4699652; doi:10.1371/journal.ppat.1005377)
Supplement: S1 Table — (DOCX) [file ppat.1005377.s001.docx]

**S1 Table: Strains and plasmids used in this work**

|  | Description | Abbreviations used |  |
| --- | --- | --- | --- |
| *Original strains transformed with pIApX2-mCherry* | | | |
| CHA [51]  CHAΔ*pscF* [52]  CHAΔ*fliC* [52]  CHAΔ*pilY1* [50]  CHAΔ*exoS*::*exoS-bla* [30] | Wild-type *P. aeruginosa* strain  CHA with an internal deletion of the *pscF* gene  CHA with a gentamycin cassette inserted within *fliC*  CHA with a transposon cassette in the *pilY1* gene  CHA with deletion of exoS and insertion of *exoS* fused to *ß-lactamase* | WT  Δ*pscF*  Δ*fliC*  Δ*pilY1*  exoS-bla |  |
| *Original strain transformed with miniCTXpX2-mCherry* | | | |
| PAK | Wild-type *P. aeruginosa* strain | PAK |  |
| *Plasmids* | | | |
| pIApX2-mCherry^a^  miniCTXpX2-mCherry^a^ | Replicative pUCP20-derived plasmid bearing pX2-mCherry fusion  Integrative mini-CTX plasmid bearing pX2-mCherry fusion |  |  |

^a^This study.
