# Supplementary figures and images for: Pseudomonas aeruginosa Transmigrates at Epithelial Cell-Cell Junctions, Exploiting Sites of Cell Division and Senescent Cell Extrusion
Source: PLoS Pathog. 2016 Jan 4;12(1):e1005377. doi: 10.1371/journal.ppat.1005377 (PMC4699652; doi:10.1371/journal.ppat.1005377)

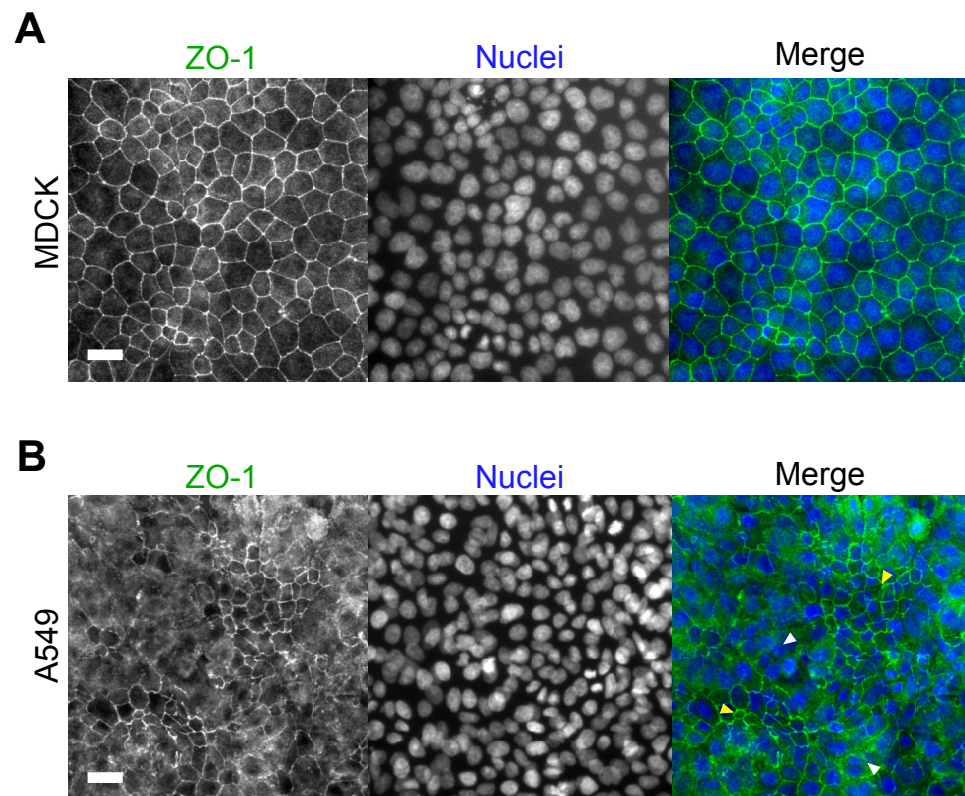

**S1 Figure**

Supplement: S1 Fig — MDCK (A) or A549 (B) cells, seeded at 2,500 cells/mm2 and cultured for 3 days, as for videomicroscopy experiments, were fixed and labeled with anti-ZO-1 antibodies (green) to highlight cell-cell junctions. Nuclei are in blue. Yellow arrowheads show ZO-1-positive junctions and white arrowheads show interrupted junctions. Scale bars: 50 μm. (PDF) [file ppat.1005377.s002.pdf]

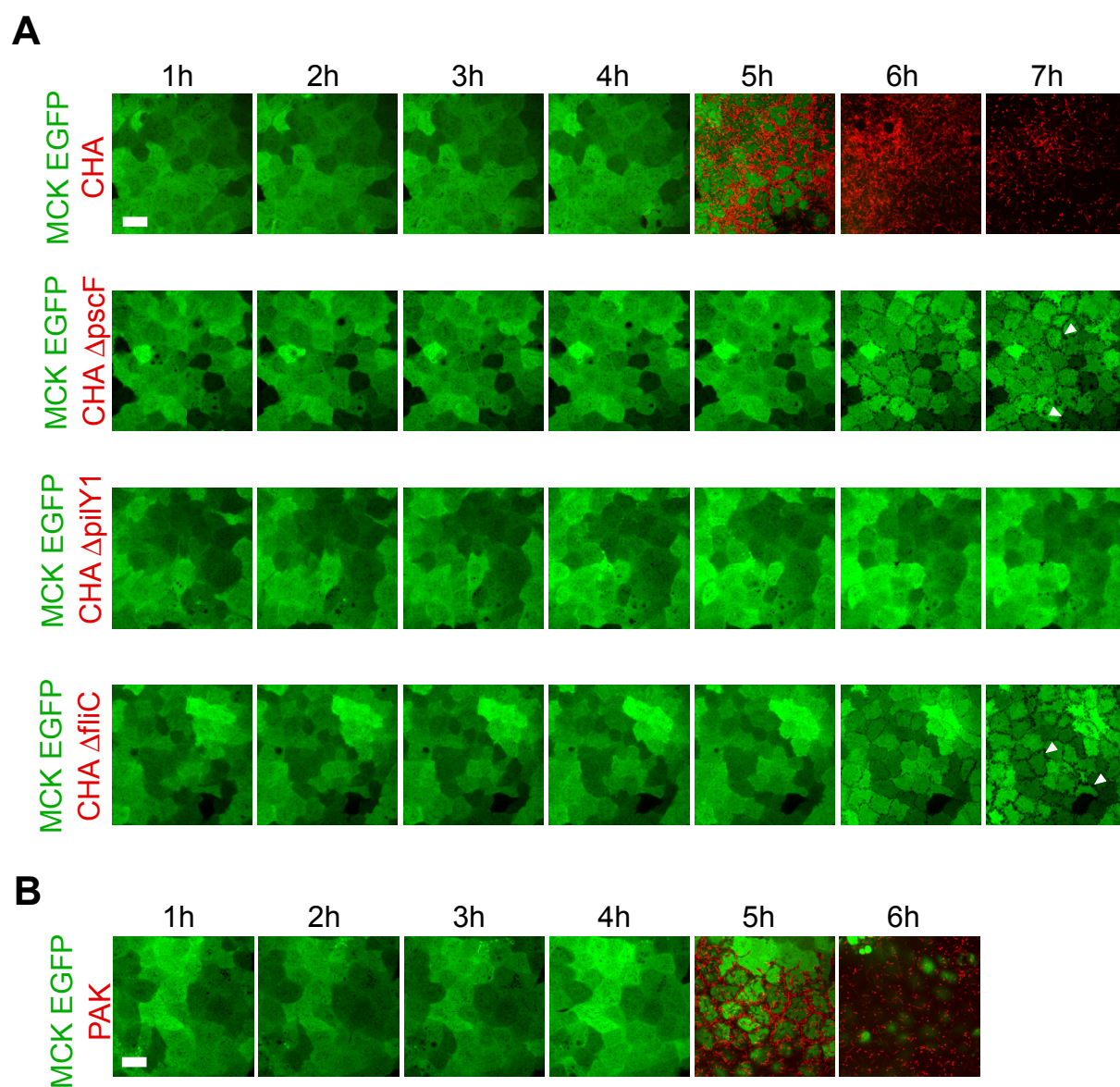

**S2 Figure**

Supplement: S2 Fig — (A,B) MDCK-EGFP monolayers were infected with various strains expressing mCherry. Merged confocal images of green and red channels, taken at the cell basal side at different time points are shown. Note that MDCK cells did not express EGFP at the same level, allowing cell individualization. (A) MDCK were infected by CHA or with CHA mutants lacking T3SS (ΔpscF), pili (ΔpilY1) or flagellum (ΔfliC). Note that at late time points, cells infected with ΔpscF or ΔfliC exhibited disrupted intercellular junctions (arrowheads), a phenotype not observed in ΔpilY1-infected cells. Quantifications of bacterial transmigration and cell retraction are shown in Fig 1C and 1E. (B) Similar experiment using PAK background. MDCK were infected with PAK. Scale bars, 20 μm. (PDF) [file ppat.1005377.s003.pdf]

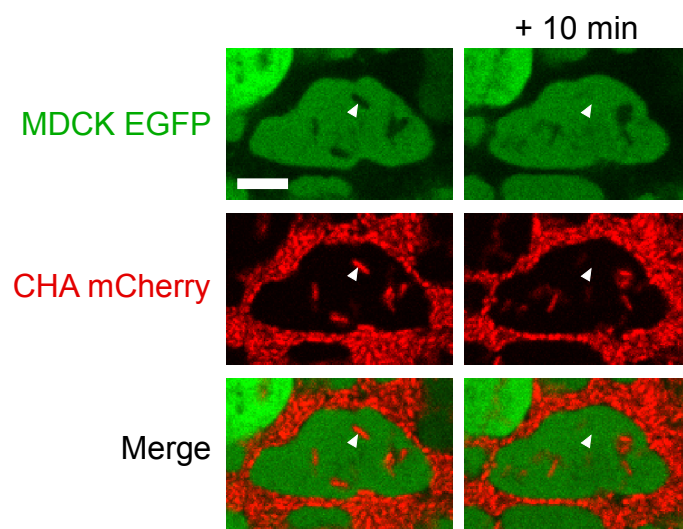

**S3 Figure**

Supplement: S3 Fig — MDCK-EGFP cells were infected with CHA-mCherry. Two successive confocal microscopy images captured at the cell’s basal side were selected to show the imprints of bacteria (arrowheads) in the cell, as monitored by the loss of green fluorescence in MDCK cytosol. Scale bars: 15 μm. (PDF) [file ppat.1005377.s004.pdf]

**A**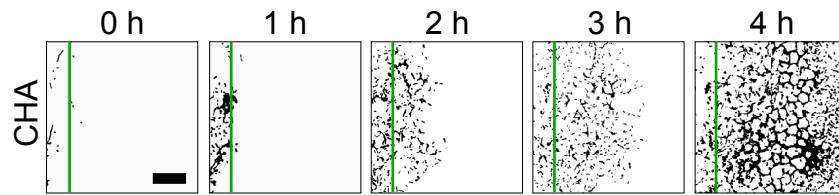**B**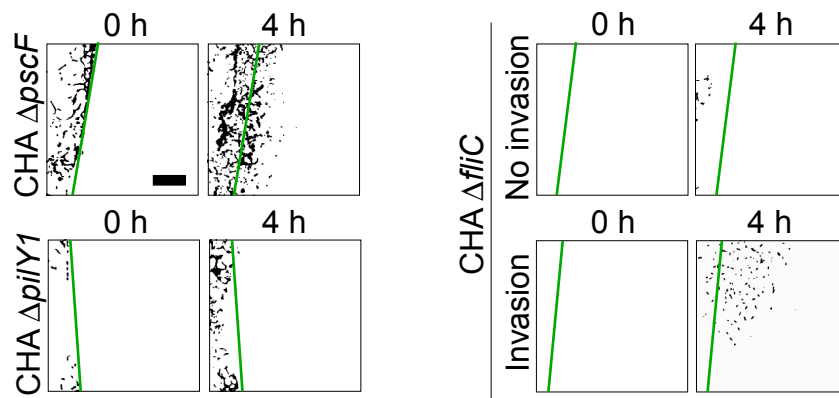**S4 Figure**

Supplement: S4 Fig — A wound was made in MDCK monolayers and bacteria were subsequently introduced in the medium. Invasion from the wound was recorded in the basal compartment by confocal microscopy at different time points, as indicated. The green lines indicate the wound edge. Scale bars: 25 μm. (A) The wounded monolayer was infected by CHA. (B) The wounded monolayers were infected with mutants lacking T3SS (ΔpscF), flagellum (ΔfliC) or pili (ΔpilY1). Note that in the ΔfliC condition, bacteria did not accumulate in the wounded area and invaded solely from specific points of the wound (“Invasion” as opposed to “No invasion”). See Fig 5 for quantifications. (PDF) [file ppat.1005377.s005.pdf]

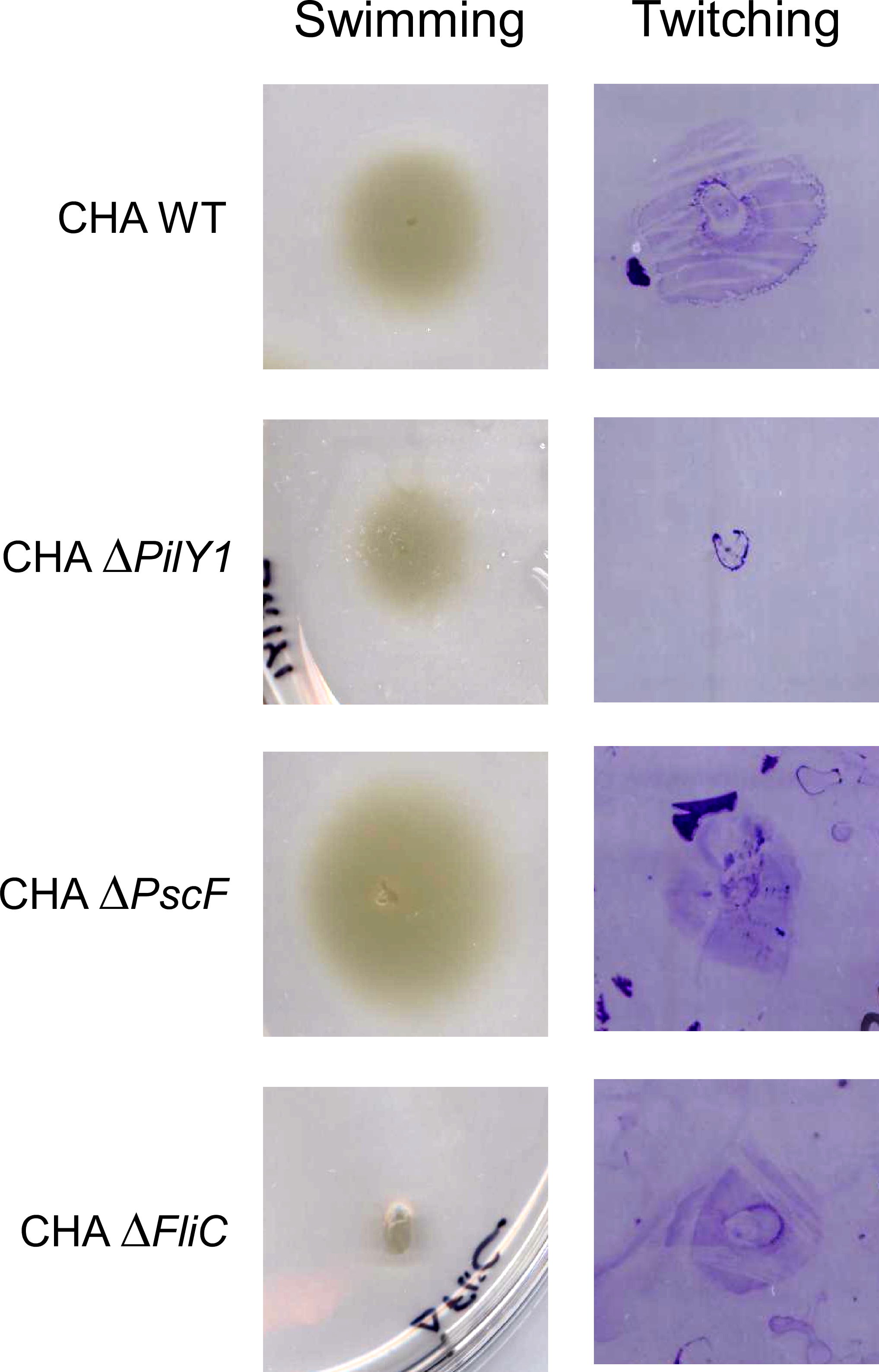

Supplement: S5 Fig — The motion types were assessed using standard procedures for the 3 mutants, compared to wild type. (TIF) [file ppat.1005377.s006.tif]
